# Conference Report: NORTH AMERICAN INTEGRATED SERVICES DIGITAL NETWORK (ISDN) USERS’ FORUM (NIUF) Gaithersburg, MD June 21–24, 1994

**DOI:** 10.6028/jres.099.071

**Published:** 1994

**Authors:** Elizabeth B. Lennon

**Affiliations:** Computer Systems Laboratory, National Institute of Standards and Technology, Gaithersburg, MD 20899-0001

## 1. Introduction

The Computer Systems Laboratory (CSL), National Institute of Standards and Technology (NIST), hosted the twenty-first North American ISDN Users’ Forum (NIUF) at its Gaithersburg, Maryland, site on June 21–24, 1994. About 175 users, implementors, and service providers of ISDN technology attended the meeting. CSL collaborated with industry in 1988 to establish the NIUF to ensure that emerging ISDN applications meet the needs of users. A Cooperative Research and Development Agreement (CRADA) with industry was established in 1991 to govern the management of the forum; as of June 1994, the CRADA had 32 signatories from industry and academia. CSL serves as chair of the forum and hosts the NIUF Secretariat. NIUF membership is open to all interested users, product providers, and service providers; meetings are held three times a year at various locations throughout North America.

## 2. The Development of ISDN Standards

International standards for ISDN support global communications for the exchange of voice, data, and image information among users, independent of any manufacturer, service provider, or implementation technology. ISDN standards are developed by the International Telecommunication Union - Telecommunication Standardization Sector (ITU-T) and in North America in particular, by the Exchange Carriers Standards Association (ECSA) accredited standards committee, T1, under the umbrella of the American National Standards Institute (ANSI).

ISDN standards provide a broad variety of options and parameters to meet many potential needs and applications. To ensure interoperability and terminal portability within the ISDN network and its attendant equipment, a uniform subset of options and parameters must be selected for implementation. Each application usually requires only a subset of total functionality available in the standards; for ISDN products and services to work together in a multivendor environment, common sets of options must be selected.

To cope with this proliferation of choices and to provide interoperable products and services which meet the needs of users, the standards specification process has been augmented to develop application profiles, implementation agreements, and conformance criteria. The NIUF addresses all of these areas.

## 3. NIUF Objectives and Structure

The NIUF seeks to achieve three principal goals:

- To promote an ISDN forum committed to providing users the opportunity to influence developing ISDN technology to reflect their needs;
- To identify ISDN applications, develop implementation requirements, and facilitate their timely, harmonized, and interoperable introduction; and
- To solicit user, product provider, and service provider participation in the process.

The actual work of the NIUF is accomplished in two workshops: the ISDN User’s Workshop (IUW) and the ISDN Implementor’s Workshop (IIW). The IUW produces application requirements which describe potential applications of ISDN and the features which may be needed. The IIW develops application profiles, implementation agreements, and conformance criteria which provide the detailed technical decisions necessary to implement an application requirement in an interoperable manner. The NIUF Executive Steering Committee coordinates the activities of the two workshops.

## 4. NIUF Achievements

Since its inception in 1988, the NIUF has achieved the following:

- 42 applications for development of application profiles are in process;
- application profiles have been completed for 17 applications;
- 15 implementation agreements have been completed; and
- 12 conformance tests have been completed.

CSL established the NIST Special Publication 823 series, Integrated Services Digital Network Technology Publications, to publish the approved implementation agreements, conformance tests, and other NIUF documents. To date five documents have been published. Copies of these documents are available for sale by the Government Printing Office, (202) 512-1800 or the National Technical Information Service, (703) 487-4650.

## 4. Highlights of June 1994 NIUF

A special one-day National Information Infrastructure (NII) Seminar focused on “Clarifying the Vision of the Information Highway.” Dr. Arati Prabhakar, NIST Director, presented the keynote address on the NII Task Force. Other presentations covered High-Performance Computing and Communications; information technology applications such as education, libraries, healthcare, and the environment; and NII application projects at NIST including the NIST NII Agent and the Advanced Technology Program (ATP) Focused Program on Information Infrastructure for Healthcare.

Tutorials presented at the June meeting included an overview of the NIUF for new users and implementors; a session on point-to-point protocol over ISDN; and a general survey of the ISDN wiring and powering work program.

Highlights from the Executive Steering Committee standing groups included an update on the new NII Working Group (NIIWG) and its agenda for meeting the needs and challenges for NII applications. The group’s charter states that the NIIWG will focus on “the requirements of efficient access to the information infrastructure, the use of standards, interoperability and the development of suitable ISDN technologies to support the NII.” The NIIWG Applications Committee and the NIIWG Architecture Committee created charters and established future projects. The NIIWG Applications Committee will re-categorize existing NIUF ISDN application profiles into appropriate NII application categories, such as healthcare. The NIIWG Architecture Committee plans to develop an ISDN portion of the NII model. The Versions — Capability and Analysis Planning meeting consisted of a presentation by a representative from Bell Atlantic on the content and schedule for National ISDN-3 specifications.

IUW working group highlights included the following: the IUW General Users’ Meeting revamped and prioritized NIUF applications, which will now be categorized as complete, inactive, or open/ongoing. The IUW and the IIW will jointly draft a letter to regional user groups to ask for assistance in championing applications within the NIUF. The Government Services Group has established a “Government” newsletter to promote local, state, federal, and international government ISDN success stories. The Private Industries Group addressed the “road blocks” that face industry organizations in the deployment of ISDN. At the Mass Market Industries Group, speakers from MFS Datanet, AT&T Global Business Communications Systems, and IBM shared their experiences with various ISDN applications. The Enterprise Network Data Interconnectivity Family demonstrated remote local-area-network (LAN) access for ISDN devices using a single B channel between seven vendors for bridging and routing, a milestone event. The Ad Hoc Group on the Simplification of ISDN Ordering, Provisioning, and Installation approved as working group stable two packages, P and Q, which were an outgrowth of ISDN Solutions ’94.

The IIW enjoyed a successful week. To maximize the effectiveness of its resources and expertise, the IIW reorganized into the following functional groups: Application Profile Team, Technical Working Group, Application Analysis, ISDN Conformance Testing, and ISDN CPE (Customer Premises Equipment) and Software Working Group.

IIW working groups reported the following activities: the Call Management Profile Team discussed open applications and determined that there are no profiles to be written at this time. At its first meeting, the Multimedia Applications and Networking Profile Team and Family announced it will focus on the development of application profiles and implementation agreements surrounding the definition, service description, network and interoperability requirements for providing end-to-end ISDN-based multimedia services. The Security and Network Management Technical Working Group and Profile Team established the foundation for contributions to NII security. The ICOT ACT 23 Working Group reviewed Basic Rate (BRI) and Primary Rate (PRI) protocol implementation conformance statements (PICS) and finalized these documents.

The ISDN CPE and Software Working Group is collecting information on ISDN-based products for inclusion in its third edition of the “Catalog of National ISDN Solutions for Selected NIUF Applications,” to be published in February 1995. Previous editions of the catalog have been distributed to thousands of end users, systems integrators, service providers, and product manufacturers and distributors.

The PBX Issues Subcommittee defined and categorized issues as they relate to PRI users and implementors, with a view toward providing technical and business solutions for these requests. These issues will be used for input to the ongoing national ISDN process. The ISDN Powering and Wiring Group received Plenary approval for certain sections of their current ISDN Powering and Wiring Guidelines (Residence and Small Businesses). Future work might include a pamphlet for ISDN Wiring and a possible new document on ISDN Wiring and Powering for High-Rise and Apartment Buildings. The CPE Compatibilities and Capabilities Profile Team heard a presentation on processing for CPE-to-CPE application interoperability between heterogeneous systems.

The closing Plenary approved three new documents and announced two documents as working group stable. Also approved were sue working group charters: National Information Infrastructure Working Group (NIIWG); NIIWG–Applications Committee; NIIWG–Architecture Committee; ISDN Security Family; Multimedia Applications and Networking Profile Team and Family; and Private Industries.

## 5. For More Information

For more information about the NIUF and its publications or to obtain conference proceedings, contact the NIUF Secretariat: Sara Caswell, Computer Systems Laboratory, National Institute of Standards and Technology, Building 223, Room B364, Gaithersburg, MD 20899-0001; telephone (301) 975-2937 or fax (301) 926-9675.

